# Exploring Fecal Microbiota Transplantation: Potential Benefits, Associated Risks, and Challenges in Cancer Treatment

**DOI:** 10.1002/cnr2.70455

**Published:** 2026-01-06

**Authors:** Aswathi Ramesh, Rajasekaran Subbarayan, Rupendra Shrestha, Pooja Narain Adtani

**Affiliations:** ^1^ Centre for Advanced Biotherapeutics and Regenerative Medicine, Faculty of Research Chettinad Hospital and Research Institute, Chettinad Academy of Research and Education Chennai India; ^2^ Centre for Herbal Pharmacology and Environmental Sustainability, Chettinad Hospital and Research Institute, Chettinad Academy of Research and Education Chennai India; ^3^ Department of Natural and Applied Sciences Nexus Institute of Research and Innovation (NIRI) Lalitpur Nepal; ^4^ Department of Basic Medical and Dental Sciences College of Dentistry, Gulf Medical University Ajman UAE

**Keywords:** cancer, fecal microbiota transplantation, gut microbiome, immunotherapy, oncogenic microbes

## Abstract

**Background:**

Fecal microbiota transplantation (FMT) has emerged as a groundbreaking strategy for modulating the gut microbiome and improving cancer treatment outcomes. This review synthesizes the current evidence on the role of FMT in oncology, focusing on its potential to enhance the efficacy of immunotherapy, restore microbiome homeostasis, and mitigate cancer‐associated complications.

**Recent Findings:**

Preclinical and clinical studies have demonstrated that FMT can reprogram the tumor microenvironment, augment immune checkpoint inhibitor responses, and reduce chemotherapy‐induced toxicity. However, risks such as pathogen transmission, immune dysregulation, and unintended microbial shifts necessitate rigorous donor screening and a personalized approach. Challenges in standardization, regulatory frameworks, and mechanistic understanding further complicate their clinical translation. Emerging innovations, including precision microbial consortia, synthetic biology, and biomarker‐driven strategies, have the potential to address these limitations.

**Conclusion:**

While FMT holds transformative potential in cancer care, its integration into oncological practice requires robust clinical validation, long‐term safety assessments, and interdisciplinary collaboration to harness its full therapeutic potential.

## 1 Introduction

Fecal microbiota transplantation (FMT) has emerged as a promising therapeutic approach in oncology, particularly for its interactions with the gut microbiome and its effects on cancer treatment outcomes. FMT involves the transfer of fecal matter from a healthy donor to the gastrointestinal tract of the recipient to restore a balanced microbial ecosystem [1]. Traditionally, FMT has been effective in treating recurrent *Clostridioides difficile* infection. Recent research has expanded its potential applications to include the modulation of the gut microbiome in patients with cancer [2]. The gut microbiome significantly influences the efficacy and toxicity of cancer therapies, including immunotherapy [3]. Studies have demonstrated that certain bacterial species can enhance response to immune checkpoint inhibitors (ICIs), whereas dysbiosis may impede treatment effectiveness [4]. Research has indicated that specific gut bacteria are associated with improved outcomes in patients undergoing immunotherapy [5]. This review focuses on the benefits, risks, and challenges of implementing FMT in cancer treatment. This has the potential to enhance the efficacy of immunotherapy by modulating gut microbiota. Possible risks include adverse reactions such as infections or unintended modulation of the immune system. These challenges include the standardization of FMT procedures, selection of appropriate donors, and understanding of long‐term effects. Understanding these aspects is crucial for integrating FMT into oncological practice and improving patient outcomes in the future.

## 2 Gut Microbiome and Cancer

The gut microbiome plays a pivotal role in cancer initiation, progression, and therapeutic response by regulating multiple physiological and immunological pathways [6]. The gut–brain axis forms an intricate communication network through which the intestinal microbiota modulates hepatic metabolism and neuroimmune signaling, both of which can influence cancer progression [7]. Microbial dysbiosis, characterized by an imbalance between beneficial and pathogenic taxa, contributes to systemic inflammation, hepatic dysfunction, and a tumor‐promoting microenvironment [8, 9]. Furthermore, microbial metabolites can affect brain function through neuroimmune and neuroendocrine pathways, indirectly influencing tumor growth and overall patient well‐being [10]. Dysbiosis disrupts immune homeostasis and promotes chronic low‐grade inflammation, which is a well‐established risk factor for tumorigenesis [11]. For instance, certain anaerobic bacteria produce genotoxic metabolites, such as hydrogen sulfide, which induce DNA damage and promote colorectal carcinogenesis [12]. Specific bacterial populations have also been associated with enhanced tumor proliferation, angiogenesis, and metastasis, highlighting the profound impact of microbiome composition on oncogenic signaling pathways.

In addition to tumor initiation, the gut microbiome critically influences the efficacy and toxicity of cancer therapies. During chemotherapy and radiotherapy, microbial communities modulate drug metabolism, systemic inflammation, and mucosal integrity [13]. Some bacteria can enzymatically degrade chemotherapeutic agents, thereby diminishing their bioavailability, whereas others produce metabolites that enhance cytotoxicity or protect the gut barrier [14]. Thus, a balanced microbiome contributes to improved therapeutic tolerance and reduced adverse effects. The microbiome profoundly shapes host immune competence during immunotherapy. The presence of specific taxa, such as *Akkermansia muciniphila* , *Bifidobacterium* spp., and *Faecalibacterium prausnitzii* , has been correlated with enhanced responsiveness to ICIs [4, 15]. Mechanistically, these microbes promote antigen presentation, increase effector T‐cell infiltration, and modulate cytokine profiles to favor antitumor immunity. Consequently, microbiome modulation through dietary interventions, probiotic supplementation, and FMT is being actively explored as an adjunct strategy to optimize treatment outcomes [16]. A deeper understanding of these microbe–host–drug interactions is essential for developing personalized microbiome‐informed therapeutic approaches. Integrating microbiome profiling into clinical oncology could enable predictive modeling of treatment response, paving the way for precision cancer therapy guided by microbial signatures.

## 3 Gut–Liver–Brain Axis

The gut microbiome plays a pivotal role in cancer progression and treatment responses, influencing various physiological processes and mechanisms [17]. The gut–liver–brain axis represents a complex communication network through which gut microbial signals modulate hepatic and neurological functions, collectively impacting tumor biology and therapeutic outcomes [7]. Dysbiosis, or microbial imbalance, can initiate systemic inflammation, alter bile acid metabolism, and impair intestinal barrier function, ultimately contributing to hepatic dysfunction and hepatocarcinogenesis [18]. Moreover, microbial metabolites, such as short‐chain fatty acids (SCFAs), indoles, and secondary bile acids, can influence the central nervous system by modulating neuroimmune pathways and neurotransmitter signaling, thereby indirectly affecting tumor growth, stress responses, and the well‐being of patients [19]. Dysbiosis can disrupt immune homeostasis, leading to chronic inflammation, which is a known risk factor for tumorigenesis [11]. For instance, specific bacterial taxa produce genotoxic metabolites, such as hydrogen sulfide, which can induce DNA damage and promote colorectal cancer. Conversely, beneficial taxa such as *Akkermansia muciniphila* and *Bifidobacterium* spp. enhance mucosal barrier function, reduce endotoxemia, and promote regulatory T‐cell balance, thereby maintaining immunological homeostasis [20]. ICIs efficacy is closely associated with the microbiome. Clinical data suggest that the presence of *A. muciniphila* , *Faecalibacterium prausnitzii* , and *Bifidobacterium longum* is correlated with enhanced immunotherapy response rates [4]. FMT from responder patients into germ‐free or antibiotic treated mice has been shown to restore antitumor immunity, confirming the causal role of the microbiome in therapy modulation. These findings emphasize the translational potential of microbiome modulation via dietary interventions, probiotics, or precision FMT to enhance therapeutic efficacy and minimize adverse effects.

Understanding the intricate interplay between the gut microbiome, tumor microenvironment (TME), and systemic axes, such as the gut–liver–brain network, is crucial for developing next‐generation microbiome‐based therapeutic strategies for cancer. Integrating microbiome profiling with multiomics and machine learning could enable the prediction of therapeutic responses and guide the personalization of FMT and probiotic interventions to improve patient outcomes.

## 4 Benefits of FMT in Cancer Patients

FMT has emerged as a potential adjunct therapy in oncology because of its ability to restore the gut microbiome balance, modulate immune responses, and enhance the efficacy of cancer treatments [21]. Research suggests that gut microbiota composition plays a pivotal role in determining patient response to immunotherapy, chemotherapy tolerance, and overall disease progression.

### 4.1 Enhancing Cancer Immunotherapy

**FIGURE 1:**
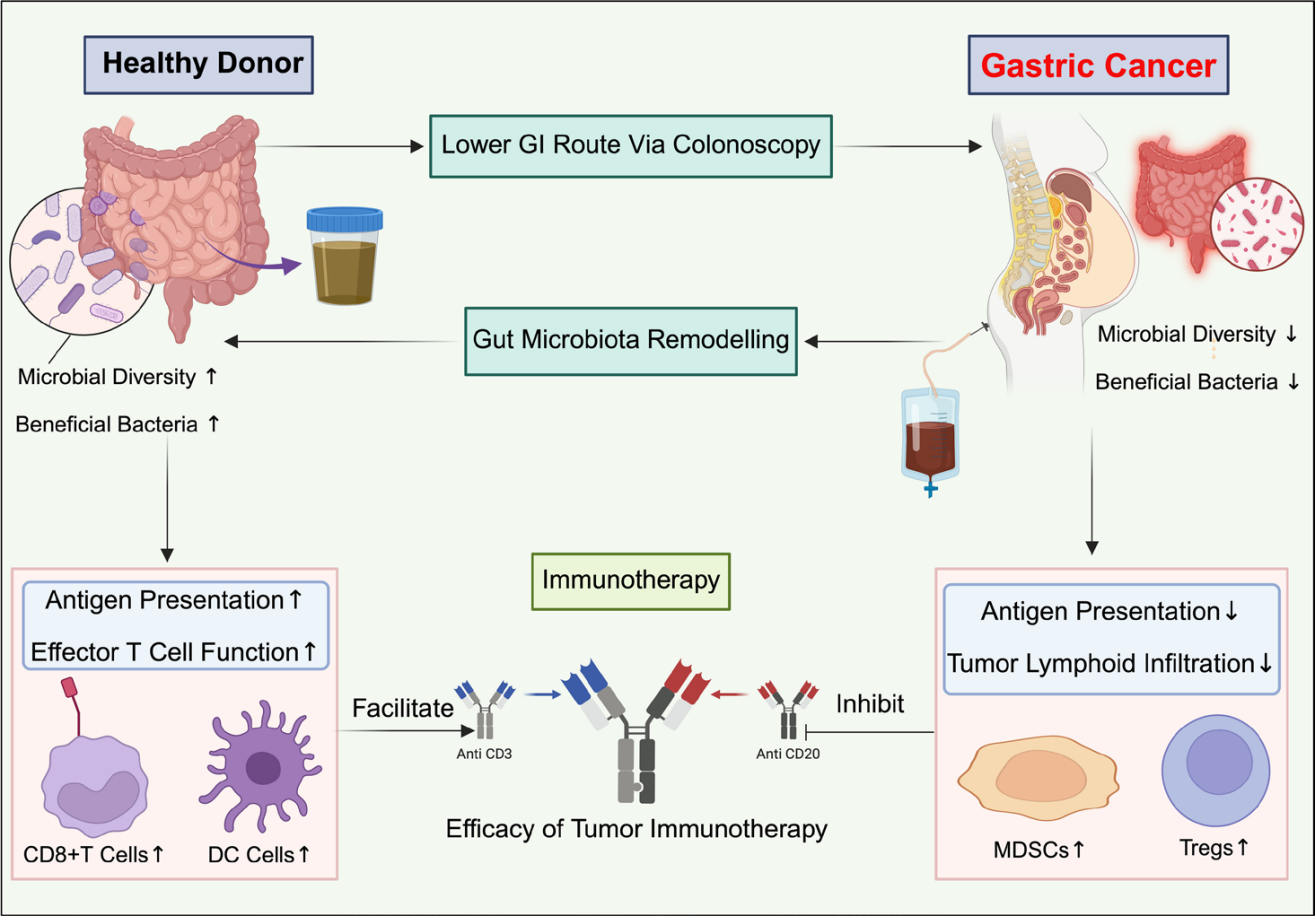
Diagram illustrating the role of FMT in enhancing tumor immunotherapy. A healthy donor's microbiota, rich in beneficial bacteria, is transferred via colonoscopy to patients with gastric cancer, promoting gut microbiota remodeling. This leads to improved antigen presentation and effector T cell function, thereby enhancing the efficacy of immunotherapy. In contrast, dysbiosis in patients with cancer suppresses immune responses by increasing the number of myeloid‐derived suppressor cells (MDSCs) and regulatory T (Treg) cells and inhibiting tumor lymphoid infiltration.

Recent studies have demonstrated that FMT can significantly improve patient responses to ICIs, including anti‐PD‐1 and anti‐CTLA‐4 therapies [22]. Researchers have identified that patients with a gut microbiome enriched in *Akkermansia muciniphila* respond better to ICIs, suggesting that microbiota composition can influence treatment success [23]. Specific bacterial taxa, including *Bifidobacterium longum* , *Faecalibacterium prausnitzii* , and *Ruminococcus* species, have been associated with favorable immunotherapy responses [24]. FMT from responders to nonresponders has been shown to enhance immune activation and tumor regression in preclinical models and early‐phase clinical trials [25]. FMT may boost immunotherapy efficacy by enhancing antigen presentation and T‐cell activation, as shown in Figure 1. Increased SCFA production regulates immune responses. This reduces the levels of proinflammatory cytokines associated with therapy resistance [26].

### 4.2 Restoration of Gut Microbiome in Cancer Patients

Cancer treatments, such as chemotherapy, radiotherapy, and prolonged antibiotic use, significantly alter the gut microbiome, leading to dysbiosis [27]. This imbalance can cause systemic inflammation, compromise the gut barrier, and contribute to treatment‐related side effects [28]. FMT reintroduces beneficial microbial diversity and restores homeostasis [29]. The gut microbiota produces key metabolites, such as SCFAs, bile acids, and tryptophan derivatives, which influence host immunity and cancer progression [30]. FMT can replenish these critical metabolites, thereby modulating systemic inflammation and immune responses.

### 4.3 Mitigating Cancer‐Associated Conditions

Chemotherapy and radiotherapy often lead to severe gastrointestinal side effects, including diarrhea, mucositis, and colitis [31]. FMT has been reported to alleviate these symptoms by restoring beneficial microbial populations and improving gut barrier integrity [32]. Clinical trials are currently investigating FMT as a treatment for immune‐related colitis in patients treated with ICIs. Cancer cachexia, characterized by severe weight loss and muscle wasting, has been linked to gut dysbiosis [33]. Certain gut microbiota compositions promote systemic inflammation via the production of LPS and proinflammatory cytokines (IL‐6 and TNF‐α) [34]. FMT has shown promise in modulating metabolic pathways, reducing systemic inflammation, and potentially alleviating cachexia‐related symptoms [35]. FMT is a promising therapeutic approach for improving cancer treatment outcomes, particularly by enhancing the immunotherapy response, restoring gut microbiome balance, and mitigating cancer‐related complications [36]. However, large‐scale clinical trials are necessary to establish standardized protocols, assess long‐term safety, and optimize patient selection for FMT interventions in oncology.

### 4.4 Unresolved Mechanistic Questions: Correlation vs. Causation in FMT Response

Although microbial taxa such as *Akkermansia muciniphila* , *Faecalibacterium prausnitzii* , and *Bacteroides fragilis* have been associated with improved ICI responses, their causality remains debatable. Many correlations may reflect an underlying immunological or metabolic state, rather than a direct microbial effect. For instance, the enrichment of *A. muciniphila* may indicate intact mucosal integrity or reduced antibiotic exposure, both of which are associated with better therapeutic response. Similarly, SCFAs, such as butyrate, exert pleiotropic effects, being anti‐inflammatory in some settings but protumorigenic in others by promoting regulatory T cells' expansion. Future research should integrate multiomics profiling (metabolomics and single‐cell immune mapping) and causality frameworks (gnotobiotic validation and microbial knockout models) to delineate the mechanistic hierarchies. Understanding whether these taxa drive or merely accompany favorable immune landscapes is crucial for the development of precision FMT.

## 5 Risks Associated With FMT in Cancer Patients

Although FMT is being increasingly investigated as an adjunct therapy for cancer treatment, it is not without risks [37]. Patients with cancer, particularly those undergoing chemotherapy, radiotherapy, or immunotherapy, often have compromised immune systems, making them more vulnerable to the adverse effects associated with microbiome manipulation [38]. The following risks should be carefully considered when implementing FMT in oncology.

### 5.1 Infection Risk and Pathogen Transmission

Despite rigorous donor screening, there is always a risk of undetected pathogens being transferred through FMT [39]. Bacterial, viral, fungal, and parasitic infections may be transmitted, posing a significant threat to immunocompromised cancer patients. Cases of *Escherichia coli* bacteremia and *Clostridium difficile* reinfection have been reported following FMT in immunocompromised patients [40]. Viral infections, including those caused by latent viruses such as cytomegalovirus (CMV) and Epstein–Barr virus (EBV), could theoretically be reactivated following FMT, although data on this are limited [41]. Similarly, FMT has been linked to the transmission of extended‐spectrum beta‐lactamase (ESBL)‐producing *Enterobacteriaceae*, a group of antibiotic resistant bacteria [42]. Patients with cancer who are already at risk of hospital‐acquired infections may experience worsened outcomes if resistant organisms colonize the gut following FMT. Despite its therapeutic potential, FMT is associated with several clinical and biosafety risks. Common short‐term adverse events include abdominal discomfort, flatulence, nausea, and mild diarrhea, which are generally self‐limiting. However, although rare, severe complications have been reported, such as *Enterobacteriaceae* or *Enterococcus* bacteremia in immunocompromised recipients and aspiration pneumonia during colonoscopic or nasojejunal administration [43]. The US FDA has also documented cases of transmission of multidrug‐resistant organisms (MDROs) and latent viral infections via inadequately screened donor stools. In addition to the risk of infection, FMT may cause unintended long‐term consequences, including the horizontal transfer of antimicrobial resistance genes, alterations in bile acid metabolism, and the potential promotion of oncogenic pathways in predisposed individuals. Immunological risks also persist because the donor microbiota may elicit unpredictable immune responses or interfere with checkpoint blockade therapy. Consequently, rigorous donor screening, standardized manufacturing, and long‐term follow‐up are indispensable to mitigate these risks and ensure safe clinical translation in oncology.

### 5.2 Immune Dysregulation and Autoimmune Activation

FMT may induce immune activation, which can trigger or worsen autoimmune disorders, particularly in patients with cancer and pre‐existing immune dysfunction [44]. Inflammatory cytokines, such as IL‐6 and TNF‐α, can be upregulated, leading to immune‐related adverse events [44]. In another study, hematopoietic stem cell transplant (HSCT) recipients were found to be particularly vulnerable, as FMT could stimulate immune responses that exacerbate graft‐versus‐host disease (GVHD) [45]. Under these conditions, donor immune cells attack the recipient tissues. Cases of severe colitis and systemic inflammation have been observed following FMT in transplant recipients.

### 5.3 Microbiome Imbalance and Dysbiosis

Although FMT aims to restore the microbiome balance, unintended shifts in microbial populations can have negative effects. Overgrowth of certain bacterial species, such as *Enterococcus faecalis* and *Klebsiella pneumoniae* , may disrupt gut homeostasis and cause inflammation [46]. Similarly, the effect of introducing a completely new microbial ecosystem into cancer patients remains poorly understood. Some studies have shown that long‐term changes in the microbiome following FMT may influence metabolism, immune function, and mental health [47].

### 5.4 Tumorigenic Potential and Oncogenic Microbes

Some gut bacteria have been implicated in tumorigenesis, raising concerns that FMT could inadvertently introduce procarcinogenic species. *Fusobacterium nucleatum* , which promotes colorectal cancer progression, has been found to be more abundant in some post‐FMT cases [48]. Certain bacterial metabolites, such as secondary bile acids and reactive nitrogen species, have been linked to DNA damage and an increased risk of cancer [49]. Long‐term oncogenic risks associated with gut microbiome alterations induced by FMT remain an area of active research.

### 5.5 Mitigation Strategies

To minimize the risks associated with FMT in patients with cancer, the following strategies should be employed: stringent donor screening and comprehensive screening for pathogens, antimicrobial resistance genes, and potentially harmful microbial strains before FMT [50]. Personalized microbiome selection using metagenomic analysis to select donor microbiomes tailored to the recipient's specific needs could improve safety and efficacy. Synthetic and next‐generation FMT, such as advancements in microbiome engineering, defined microbial consortia, and stool‐derived microbial products, may offer safer alternatives to traditional FMT [51]. Therefore, long‐term monitoring is required. Post‐FMT surveillance should include microbiome profiling, immune response assessment, and monitoring of cancer progression. Although FMT holds promise as a novel therapeutic approach for cancer treatment, the potential risks must be carefully considered, particularly in immunocompromised patients. Future research should focus on refining the donor selection criteria, developing safer microbiome‐based therapies, and elucidating the long‐term consequences of microbiota modulation in patients with cancer. Reported adverse events following FMT include transient gastrointestinal discomfort (abdominal cramping, bloating, and flatulence), mild fever, and self‐limited diarrhea [52]. Rare but severe complications, such as bacteremia, aspiration during colonoscopic administration, and exacerbation of inflammatory bowel disease, have also been documented. In immunocompromised patients, opportunistic infections caused by multidrug‐resistant *E. coli* or *Enterococcus* species have been reported, underscoring the need for vigilant monitoring and robust infection control protocols [53]. Overall, the incidence of serious adverse events remains below 5% in most controlled studies, suggesting that FMT is generally safe when patients are appropriately screened and monitored.

## 6 Challenges of Implementing FMT in Cancer Care

Despite its growing potential in oncology, FMT faces significant barriers to clinical implementation [54]. These challenges encompass donor selection, standardization, regulatory frameworks, and mechanistic understanding, all of which must be addressed to optimize the safety and efficacy of FMT in patients with cancer.

### 6.1 Donor Selection and Screening

**FIGURE 2:**
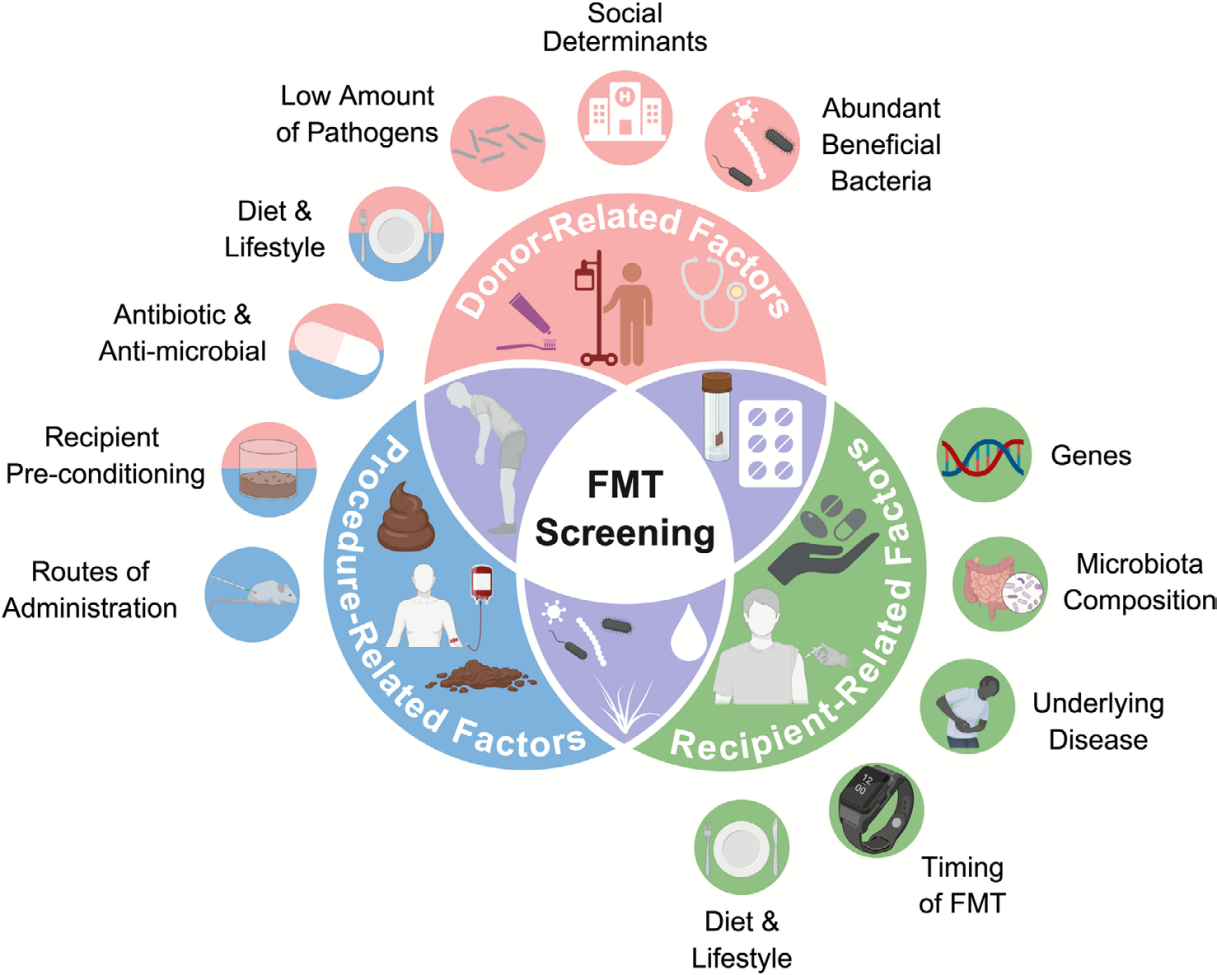
FMT screening factors: donor‐related factors (red section) include medical history, infections, gut microbiota composition, lifestyle, and dietary habits, all of which affect the quality of the transplanted microbiota. Procedure‐related factors (blue section) include sample preparation and mode of administration (e.g., oral capsules, colonoscopy, and enema). Recipient‐related factors (green section) include genetic predisposition, gut microbiota diversity, immune response, and preexisting conditions that influence the success and risks of FMT.

One of the primary challenges of FMT is ensuring that donor microbiomes are safe and beneficial for patients with cancer, particularly those with compromised immune systems. Figure 2 shows the screening factors for FMT. Despite rigorous screening, undetected pathogens in donor stool pose a significant risk, particularly in immunosuppressed patients undergoing chemotherapy or immune checkpoint inhibition [55]. Cases of multidrug‐resistant *E. coli* transmission via FMT have been reported, underscoring the need for enhanced screening protocols [40]. Not all microbiomes are beneficial for every cancer patient; an FMT that benefits one individual may have neutral or even adverse effects on another [56]. Studies suggest that specific bacterial taxa, such as *Bacteroides fragilis* and *Faecalibacterium prausnitzii* , may enhance immunotherapy responses, whereas other strains may exacerbate inflammatory conditions [23]. The concept of a “universal” microbiome donor, similar to universal blood donors, is still under investigation [57]. The current study aimed to identify the microbial composition that maximizes FMT efficacy while minimizing risks. One of the key challenges in donor screening is the detection of asymptomatic carriers of pathogens such as *Enterobacteriaceae*, *Clostridioides difficile*, *Campylobacter*, and enteric viruses (e.g., norovirus and adenovirus). Clinically healthy donors may harbor antimicrobial resistance genes or latent viral infections that evade standard diagnostic assays. Studies have indicated that only 10%–15% of potential donors typically qualify after complete screening, which includes medical history, serological testing, stool pathogen screening, and antibiotic resistance profiling [58]. This low qualification rate underscores the difficulty of maintaining consistent donor availability and highlights the need for synthetic, donor‐independent alternatives.

### 6.2 Personalization and Standardization

The clinical use of FMT in oncology is hindered by the lack of standardized protocols and the need for personalized approaches [36]. FMT can be delivered in different forms, including fresh stool, frozen stool, encapsulated formulations, and lyophilized powders [59]. The efficacy of these different preparations in cancer patients remains poorly understood, and there is no consensus on the best method of administration. Patients with cancer exhibit significant heterogeneity in their gut microbiome composition. Current FMT practices often do not consider individual microbiome profiles, which leads to variable responses. The optimal frequency, dosage, and timing of FMT relative to cancer treatments, such as immunotherapy, remain unclear [60]. Overdosing or inappropriate timing may result in transient dysbiosis rather than long‐term microbiome restoration.

### 6.3 Regulatory and Ethical Concerns

However, FMT is not yet fully regulated as a cancer therapy, which creates uncertainty about its clinical applications. In many countries, FMT is only approved for recurrent *Clostridium difficile* infections and is not formally recognized as a treatment for cancer‐related microbiome imbalance [54]. Regulatory agencies, including the Food and Drug Administration (FDA), categorize FMT as an investigational drug, limiting its widespread clinical application [61]. In patients with late‐stage cancer, experimental treatments such as FMT must balance potential benefits with unknown risks. Informed consent is challenging, especially given the limited long‐term safety data on FMT in oncology. Stool banking and FMT access are currently limited to a few specialized centers, making the therapy inaccessible to many cancer patients [62]. Ethical concerns have arisen regarding the commercialization of human stool for therapeutic use.

### 6.4 Limited Mechanistic Understanding

Although FMT has shown promise in modulating immune responses and improving treatment outcomes, significant gaps remain in our understanding of its precise mechanisms in cancer care. The interactions among FMT, gut microbiota, and the TME remain poorly understood [63]. While some studies suggest that FMT can enhance antitumor immunity, others indicate that microbial shifts can promote tumor growth under certain conditions [64]. The interaction between FMT‐derived microbes and the host immune system, metabolism, and systemic inflammation remains an active area of research [65]. However, the long‐term effects of microbiome modification on cancer progression and recurrence remain unknown. While FMT enhances the response to ICIs, its interactions with other cancer treatments, such as chemotherapy and radiotherapy, require further investigation [66]. Certain bacterial metabolites, such as bile acids and tryptophan derivatives, may influence drug metabolism and toxicity, necessitating caution in their clinical application [67]. Despite its potential in cancer care, FMT faces several implementation challenges, including donor screening limitations, lack of personalization, regulatory hurdles, and incomplete mechanistic understanding [62]. Addressing these barriers will require rigorous clinical trials, refined microbial selection techniques, and standardized protocols to ensure the safety and efficacy of FMT in oncology patients. With advances in research, next‐generation microbiome‐based therapies, such as defined microbial consortia and engineered probiotics, may provide more controlled and targeted alternatives to traditional FMT.

## 7 Current Evidence and Clinical Studies on FMT in Cancer Therapy

FMT is emerging as a potential therapeutic approach in oncology, particularly for modulating the gut microbiome to enhance the response to cancer treatment [54]. Although the initial findings are promising, most of the evidence comes from preclinical models and early‐phase clinical trials. This section reviews the current research, including preclinical studies in animal models and ongoing clinical trials assessing FMT's impact on cancer therapy. Several preclinical studies using mouse models have demonstrated that FMT influences tumor progression and enhances the response to cancer therapies, particularly ICIs.

### 7.1 FMT Enhancing Response to Immunotherapy

A study showed that FMT from melanoma patients who responded well to anti‐PD‐1 therapy, when administered to germ‐free or antibiotic treated mice, improved their response to ICIs [5]. The study revealed that beneficial microbial species, such as *Akkermansia muciniphila* and *Bifidobacterium* spp., promote T‐cell infiltration into tumors and enhance antitumor immunity. In addition to *Akkermansia* and *Faecalibacterium*, *Bacteroides* species are major components of transplanted microbial communities [68]. Studies have indicated that *Bacteroides fragilis* and *Bacteroides thetaiotaomicron* enhance antitumor immunity by producing polysaccharide A, which modulates dendritic cell maturation and promotes Th1 immune responses [69]. Moreover, the *Bacteroides* genus has been shown to restore gut homeostasis following chemotherapy‐induced dysbiosis, although excessive abundance may also drive inflammation in some contexts [70]. Thus, including and regulating *Bacteroides* populations during FMT is critical for balancing therapeutic efficacy and safety.

### 7.2 Role of FMT in Chemotherapy and Radiotherapy Sensitization

Another study has demonstrated that gut microbiota plays a crucial role in modulating responses to chemotherapy. Mice treated with broad‐spectrum antibiotics showed impaired responses to cyclophosphamide, suggesting that microbe‐derived metabolites contribute to immune activation [71]. Similar findings have been reported for radiotherapy, in which microbial diversity influences radiation‐induced inflammation and DNA damage repair.

### 7.3 Tumor Suppressive vs. Tumor‐Promoting Effects

While FMT has been shown to enhance antitumor immunity, some studies have suggested that an inappropriate microbiome shift can promote tumorigenesis [72]. Researchers have found that *Fusobacterium nucleatum* transferred via FMT accelerates colorectal cancer growth by suppressing immune responses and promoting chemoresistance [73]. These findings underscore the need for the precise selection of microbes in FMT protocols to avoid the introduction of tumor‐promoting bacteria. The translation of FMT into clinical oncology is still in its early stages; however, multiple trials are investigating its safety and efficacy in various cancers, particularly when it is combined with immunotherapy.

Emerging evidence indicates that FMT can reprogram the tumor TME by restoring microbial homeostasis and reshaping host immune dynamics. FMT introduces a balanced consortium of commensal microbes that enhance antigen presentation, stimulate dendritic cell maturation, and increase cytotoxic CD8^+^ T cell infiltration within the TME. These changes counteract immunosuppressive signaling, reduce regulatory T cell activity, and downregulate inhibitory checkpoints, such as PD‐L1 expression. Additionally, microbe‐derived metabolites, including butyrate and propionate, modulate epigenetic regulation and cytokine profiles, promoting an anti‐inflammatory yet immunostimulatory milieu that favors tumor suppression. Such immune reprogramming not only potentiates the efficacy of ICIs but also improves treatment tolerance and reduces therapy‐induced mucosal injury. In the context of therapy, the gut microbiome significantly affects the efficacy and toxicity of anticancer treatment. During chemotherapy and radiotherapy, microbial composition determines the host's drug metabolism and susceptibility to mucositis and systemic inflammation. Certain microbial enzymes metabolize chemotherapeutic agents, thereby altering their bioavailability and pharmacodynamics. Furthermore, a healthy gut ecosystem is essential for maintaining intestinal integrity, which is frequently compromised during cytotoxic treatment and predisposes patients to infections and inflammation.

### 7.4 Completed Clinical Studies on FMT in Cancer Patients

These studies suggest that FMT helps overcome resistance to ICIs by restoring beneficial microbial taxa associated with antitumor immunity (Table 1).

**TABLE 1:** Completed FMT clinical trials in patients with cancer.

S. no.	Cancer type	Intervention	Key findings	References
1	Melanoma	FMT + Pembrolizumab	Partial and complete responses observed; increased T‐cell activation	[74]
2	Gastro‐esophageal cancer	FMT from overweight or obese donor to Cachectic patients with advanced stage cancer	FMT from a healthy obese donor prior to first‐line chemotherapy did not affect cachexia, but may have improved response and survival in patients with metastatic gastroesophageal cancer	[75]
3	Liver cirrhosis	HBeAg after long‐term antiviral therapy were given FMT	FMT can effectively restore gut microbial diversity and function disrupted by antibiotic therapy	[76]
4	Hepatic encephalopathy	Rational stool donor improves hepatic encephalopathy	Increased microbial diversity and beneficial taxa. Study concluded that FMT from a rational donor is safe and may reduce hospitalization	[77]
5	Melanoma	FMT promotes response in immunotherapy‐refractory melanoma patients	The gut microbiome has been shown to influence tumor response to anti‐PD‐1 immunotherapy in preclinical mouse models and observational patient cohorts	[78]

Recent Phase I/II studies have begun to translate FMT into oncological settings, particularly for restoring responsiveness to ICIs. Studies have demonstrated that FMT from ICI responders induces objective responses in 20%–30% of previously refractory melanoma patients, accompanied by the expansion of cytotoxic CD8^+^ T cells and decreased infiltration of myeloid suppressor cells [79]. However, these small, nonrandomized trials (*n* = 10–15 per arm) were limited by donor variability, short follow‐up (< 12 months), and a lack of mechanistic biomarkers predictive of sustained benefit. In contrast, studies on metastatic renal cell carcinoma [80] and NSCLC have shown transient immune activation without a significant clinical response, suggesting that tumor type, antibiotic exposure, and baseline microbiome composition influence therapeutic outcomes [81]. Collectively, early phase trials have provided encouraging yet heterogeneous results, underscoring the urgent need for randomized, donor‐matched, and longitudinally monitored FMT studies to validate its reproducibility and safety in cancer cohorts.

### 7.5 Ongoing Clinical Trials Exploring FMT in Cancer Patients

Several clinical trials are currently being conducted to investigate the role of FMT in cancer treatment. The first trial focused on FMT and immunotherapy synergy (Clinical Trial: NCT04130763) [82], in which the evaluation aimed to determine whether FMT from ICI responders can improve immunotherapy outcomes in melanoma patients who have failed initial anti‐PD‐1 therapy. Another clinical trial examined the potential of FMT to counteract antibiotic induced dysbiosis in patients with cancer (Clinical Trial: NCT04264975) [83], specifically investigating whether FMT can restore gut microbiome balance in patients with lung cancer who received antibiotics before immunotherapy. Finally, research is being conducted on FMT for preventing chemotherapy‐induced toxicity (Clinical Trial: NCT04577729) [84], assessing whether it can reduce chemotherapy‐induced diarrhea and gut inflammation in patients with colorectal cancer.

Despite these promising results, several challenges remain in the translation of FMT into routine cancer therapy. The lack of standardization in donor selection and microbial composition makes it difficult to replicate the results across different studies [58]. However, long‐term safety concerns, particularly those related to the introduction of tumor‐promoting bacteria, require further evaluation. Regulatory hurdles remain, as FMT is currently only approved for recurrent *Clostridium difficile* infections in many countries [85]. The next step is to develop defined microbial consortia rather than whole‐stool FMT to ensure safety and precision. Large‐scale randomized controlled trials are recommended to confirm the efficacy and long‐term benefits of this treatment for various types of cancer. Exploring engineered probiotics and postbiotics as alternatives to traditional FMT [86]. Emerging evidence from preclinical and early phase clinical trials suggests that FMT holds promise for enhancing cancer treatment outcomes, particularly through immunotherapy. However, rigorous research is still needed to standardize FMT protocols, identify optimal microbial compositions, and evaluate the long‐term safety of FMT in patients with cancer. As research advances, FMT may become a key tool in microbiome‐based precision medicine for oncology.

## 8 Future Directions and Challenges in Clinical Translation

As the therapeutic potential of FMT in oncology has gained recognition, research efforts have shifted toward refining its safety, precision, and efficacy. Current innovations aim to move beyond traditional whole stool transplantation toward more controlled, personalized microbiome‐based interventions. This section explores four key future directions for FMT Success: precision FMT, combination therapies, safety improvements, and biomarkers. One of the biggest challenges of FMT in cancer therapy is the unpredictability of outcomes owing to the complexity and variability of the gut microbiota. Precision FMT aims to develop standardized, personalized microbiota‐based treatments instead of relying on donor stool from unknown sources [62].

In the development of personalized microbiota consortia, researchers are working on defined microbial consortia composed of carefully selected groups of beneficial bacterial strains that can be standardized and tailored to individual patients rather than transferring an entire gut microbiome. Studies have identified specific bacterial taxa, such as *Akkermansia muciniphila* , *Faecalibacterium prausnitzii* , and *Bifidobacterium* spp., that enhance immune responses and may improve ICI outcomes [87]. Personalized FMT can be based on a patient's pre‐existing microbiome profile, ensuring compatibility and maximizing therapeutic effects. The use of Synthetic Microbiota or Next‐Generation Probiotics (NGPs), such as laboratory‐engineered bacterial communities designed to mimic a healthy gut microbiome, is emerging as an alternative to traditional FMT. NGPs differ from conventional probiotics (*Lactobacillus* and *Bifidobacterium*) by including novel strains with enhanced therapeutic potential [88], such as *Clostridium butyricum* (a producer of SCFAs) and *Eubacterium hallii* (which modulates bile acid metabolism). Microbial engineering allows the creation of bacteria that produce anti‐inflammatory metabolites, immune‐boosting molecules, and enzymes that detoxify harmful compounds in the gut [89]. However, FMT alone may not be sufficient for long‐term microbiome stability in patients with cancer. Future approaches should focus on combining FMT with complementary interventions to enhance its effectiveness and maintain a healthy gut ecosystem. FMT with Prebiotics and Dietary Interventions: Prebiotics can help maintain the colonization of beneficial microbes post‐FMT [90]. In addition, dietary interventions, such as fiber‐rich or ketogenic diets, are being investigated for their potential to enhance immunotherapy responses by modulating the microbiome [91]. Finally, postbiotic bioactive compounds produced by beneficial microbes are being explored as adjunct therapies to reinforce microbiome stability without the need for live bacteria.

Several defined microbial consortia and live biotherapeutic products have recently received regulatory approval or are in advanced clinical development, representing a shift toward standardized, donor‐independent microbiome therapies. For instance, *REBYOTA (RBX2660)* and *VOWST (SER‐109)* have been approved for preventing recurrent *Clostridioides difficile* infections. These products consist of well‐characterized spore‐forming bacterial communities that ensure consistent composition and product safety. *VE303* (Vedanta Biosciences) is another rationally defined bacterial consortium currently in Phase II development, formulated with eight nonpathogenic *Clostridia* strains that restore colonization resistance and mucosal immunity. Although developed for *Clostridioides difficile*, these innovations establish a regulatory and mechanistic framework that could accelerate the development of microbiome‐based therapies for cancer.

FMT combined with engineered bacteria can be designed to directly produce therapeutic compounds, such as ICIs or anti‐inflammatory cytokines, in the gut. Engineered strains of *E. coli* and *Lactococcus lactis* have been tested for their ability to deliver cancer‐fighting molecules [92]. This approach could provide a more controlled and sustained therapeutic effect than traditional FMT. Integration with Cancer Therapies: FMT has been investigated for its ability to enhance immunotherapy responses by replenishing beneficial gut bacteria that interact with the host immune system. Some studies have suggested that FMT can reduce chemotherapy‐induced gut toxicity by restoring gut barrier integrity and reducing inflammation. Future trials should explore whether sequential FMT treatments (before, during, and after therapy) can further optimize cancer treatment outcomes.

### 8.1 Safety Improvements: Enhancing the Reliability of FMT

Despite its promise, FMT carries inherent risks, including pathogen transmission and unintended alterations to the microbiome. Future innovations will aim to improve the safety, reproducibility, and regulatory approval of clinical use. Traditional FMT relies on donor stool and requires rigorous screening for bacterial, viral, and parasitic infections. However, rare cases of multidrug‐resistant bacterial transmission have raised concerns [93]. New techniques, such as metagenomic sequencing, enable more precise donor screening by analyzing the complete microbial composition of stool samples. Standardized stool banks have been established to ensure high‐quality and consistent FMT materials [94]. Some initiatives are working to create a library of well‐characterized FMT donors to enable better donor matching based on patient microbiome profiles. Researchers are currently investigating synthetic microbiomes that are free of potential pathogens and unwanted bacteria. CRISPR‐based approaches allow the selective removal of harmful bacteria from FMT preparations before transplantation.

### 8.2 Biomarkers for FMT Success: Predicting Treatment Response

One major challenge in FMT research is that not all cancer patients respond equally to treatment. Identifying biomarkers could help predict who will benefit from FMT and guide personalized microbiome‐based interventions.

#### 8.2.1 Microbial Biomarkers

Studies have suggested that higher levels of beneficial bacteria, such as *Akkermansia muciniphila* and *Bifidobacterium* spp., correlate with better responses to immunotherapy [23]. Conversely, the presence of pathogenic bacteria, such as *Fusobacterium nucleatum* , has been linked to poor cancer prognosis and treatment resistance.

#### 8.2.2 Host Immune Biomarkers

Certain immune cell signatures (e.g., high CD8+ T‐cell infiltration) may indicate a better response to microbiome‐based therapy [95]. Cytokine profiling (e.g., IL‐10 and TNF‐α levels) is being explored as a means of monitoring the efficacy of FMT in patients with cancer.

#### 8.2.3 Metabolomic Biomarkers

The gut microbiota produces various metabolites (such as SCFAs and bile acids), which influence tumor immunity and inflammation. Monitoring microbial metabolites in the blood or stool could provide early indicators of FMT success or failure [96].

#### 8.2.4 Predictive Machine Learning Models

AI‐based tools are being developed to analyze gut microbiome sequencing data and predict patient responses to FMT [97]. These models integrate microbiome, immune, and metabolomic data to provide personalized treatment recommendations.

Donor‐independent microbiome therapeutics are rapidly emerging as safer and more standardized alternatives to traditional FMT. These include synthetic microbial ecosystems, spore‐based preparations, and cultured bacterial consortia, all of which eliminate donor variability and reduce the risk of pathogen transmission. Engineered microbiota, such as the *Microbial Ecosystem Therapeutics (MET)* platform, use precisely defined microbial strains to restore metabolic and immune homeostasis without the need for whole stool transplantation. Such innovations are expected to transform microbiome therapy into a reproducible, regulatory‐compliant, and scalable approach for integration into oncology.

## 9 Conclusions

FMT represents a paradigm shift in oncology, introducing the gut microbiome as a modifiable therapeutic axis to enhance cancer treatment outcomes. Accumulating clinical and preclinical evidence supports the capacity of FMT to augment ICI efficacy, particularly in melanoma and gastrointestinal cancers, through the enrichment of beneficial taxa, such as *Akkermansia muciniphila* , *Bifidobacterium* spp., and *Faecalibacterium prausnitzii* . Beyond immunomodulation, FMT has the potential to alleviate treatment‐related dysbiosis, chemotherapy‐induced toxicity, and metabolic imbalances associated with cancer cachexia.

However, their clinical translation has considerable hurdles. Risks such as pathogen transmission, introduction of tumorigenic microbial species, horizontal gene transfer, and immune‐mediated adverse effects necessitate stringent donor screening, rigorous product standardization, and careful post‐treatment surveillance. Furthermore, the absence of globally harmonized regulatory frameworks and variability in stool preparation and administration methods impede reproducibility and widespread clinical acceptance.

Future research should focus on establishing precision microbiome therapeutics, including synthetic microbial consortia, defined bacterial cocktails, and engineered probiotics, to overcome donor dependency and enhance safety. The integration of FMT with prebiotics, dietary modulation, and metabolite‐producing microbial therapies could synergistically stabilize gut ecology and potentiate anticancer efficacy. Advances in biomarker discovery, multiomics integration, and machine learning‐based response prediction are pivotal for patient stratification and personalized treatment design. Ultimately, translating FMT into a standardized, mechanism‐driven, and ethically regulated intervention requires collaborative, multicenter Phase II/III trials to assess long‐term efficacy and safety. By bridging innovation with evidence‐based science, FMT has the potential to redefine precision oncology by transforming the gut microbiome from a passive participant into a central, targetable regulator of cancer therapy success.

## Author Contributions

**Rajasekaran Subbarayan and Rupendra Shrestha:** conceptualization. **Aswathi Ramesh**, **Rajasekaran Subbarayan**, and **Rupendra Shrestha:** formal analysis, investigation, writing – original draft, writing – review editing, and visualizations. **Pooja Narain Adtani:** formal analysis, writing – original draft, writing – review editing. All authors approved the final version of the manuscript after reviewing their contributions and providing critical feedback.

## Funding

The authors have nothing to report.

## Ethics Statement

The authors have nothing to report.

## Consent

The authors have nothing to report.

## Conflicts of Interest

The authors declare no conflicts of interest.

## Data Availability

Data sharing not applicable to this article as no datasets were generated or analysed during the current study.
